# Prevalence and Determinants of Hookah Smoking Among the Youth: A Scoping Review

**DOI:** 10.7759/cureus.79952

**Published:** 2025-03-03

**Authors:** Tony Jehi, Pamela Serban, Anupama Joshi, Dania Matta, Archana Sharma, Matthew Mutchler

**Affiliations:** 1 School of Public Health &amp; Health Sciences, CSUDH (California State University, Dominguez Hills), Carson, USA; 2 School of Public Health, Loma Linda University, Loma Linda, USA; 3 Department of Child Development, CSUDH (California State University, Dominguez Hills), Carson, USA; 4 School of Undergraduate and Graduate Studies, National University of Natural Medicine, Portland, USA; 5 Department of Health Sciences, CSUDH (California State University, Dominguez Hills), Carson, USA

**Keywords:** determinants, hookah smoking, prevalence, scoping review, youth

## Abstract

Hookah smoking is highly prevalent and has been rising in popularity among young people worldwide. Yet, no recent and comprehensive reviews have been published to assess the prevalence and identify the main predictors of hookah use. We have thus carried out a comprehensive scoping review to consolidate and review the existing evidence for the prevalence and main determinants of hookah smoking among youth. A comprehensive literature search was thus conducted utilizing various databases including ScienceDirect, Scopus, Embase, and MEDLINE to identify relevant studies. To be incorporated in this review, studies had to include individuals below the age of 25, measure the prevalence of hookah smoking, or examine the determinants of hookah smoking. The review showed that hookah is mostly prevalent among youth from the Middle Eastern region, USA, South Asia, and various European countries. It also identified the main determinants of hookah smoking, which include age, the male gender, socioeconomic status, geographic region, other forms of substance use, knowledge, beliefs, and attitudes toward hookah smoking, sensation seeking, having friends and/or family members who smoke hookah, social acceptability, intention, accessibility, and lack of enforcement of prohibiting laws. The main determinants of hookah use include having friends and/or family members who smoke hookah, social acceptability, attitude, accessibility, and lack of enforcement of prohibiting laws. Public health authorities, educators, and other stakeholders should implement educational interventions to enhance the knowledge level on hookah smoking’s harm and addiction and should target not only the individuals but also the family and the social environment.

## Introduction and background

Hookah smoking, a method of inhaling tobacco smoke through a water pipe, is highly prevalent among youth [1]. According to the Centers for Disease Control and Prevention (CDC), almost 8% of high school students reported using hookah to smoke tobacco in 2018 in the United States [1]. It is suggested by literature that youth initiate hookah smoking during their early to mid-teen years [2-15]. This prevalence is concerning since according to the American Lung Association, hookah smoking is associated with various acute and chronic health effects. Short-term use leads to decreased pulmonary function and increased blood pressure and heart rate. Long-term effects include increased risk of chronic conditions such as heart disease, impaired pulmonary function, chronic obstructive pulmonary disease, along with a variety of cancers [16].

It is alarming that although youth are not aware of these harmful risks, there are very few public health interventions that have ever targeted it among this population. The US Food and Drug Administration (FDA) does not have strict regulations for the production, distribution, marketing, and sales of hookahs [17]. Assessing the prevalence and main predictors of hookah smoking is thus highly critical as it would highlight the urgency of conducting family- and policy-wide interventions.

Even though various reviews have been published to summarize the overall evidence on the various correlates of hookah smoking including affordability and accessibility, substance, culture, mental health, positive social appearance, and misconceptions about its lack of health impacts, these reviews did not focus on youth [18-21] or were confined to a specific region [22,23].

We have thus conducted a comprehensive scoping review to consolidate and review existing evidence on the global prevalence and main determinants of hookah smoking among youth. We hypothesize that hookah use among youth will mostly be determined by interpersonal/relationship factors.

## Review

Methods

Search Strategy and Inclusion Criteria

The search was conducted based on Preferred Reporting Items for Systematic Reviews and Meta-Analyses (PRISMA) [24]. A comprehensive electronic search was carried out in Google Scholar, ScienceDirect, Scopus, Embase, and MEDLINE to identify relevant studies published until September 2023. Articles were also extracted from the reference lists of the identified studies.

The following key terms were searched: (1) (("hookah" OR "shisha" OR "waterpipe" OR "narghile" OR "argileh") AND ("youth" OR "students" OR "college students" OR "children") AND ("predictors" OR "factors" OR "determinants" OR "correlates")); (2) (("hookah" OR "shisha" OR "waterpipe" OR "narghile" OR "argileh") AND ("youth" OR "students" OR "college students" OR "children") AND ("prevalence" OR "use")).

To be incorporated in this review, studies had to (i) be published in peer-reviewed journals; (ii) be written in English; (iii) include youth or individuals below the age of 25; (iv) measure the prevalence of hookah smoking; and (v) examine the determinants of hookah smoking. We excluded investigations that included a study population with an average age exceeding 24 years, did not measure the prevalence or determinants of hookah smoking, and were review studies. The search was restricted to observational studies; no randomized controlled trials were included in the search.

*Screening and Data Extraction* 

T.J. and P.S. independently screened the retrieved abstracts and full texts and evaluated them for inclusion using EndNote X7 [25]. Disagreements between the two authors were resolved by discussion with D.M.

**Figure 1:**
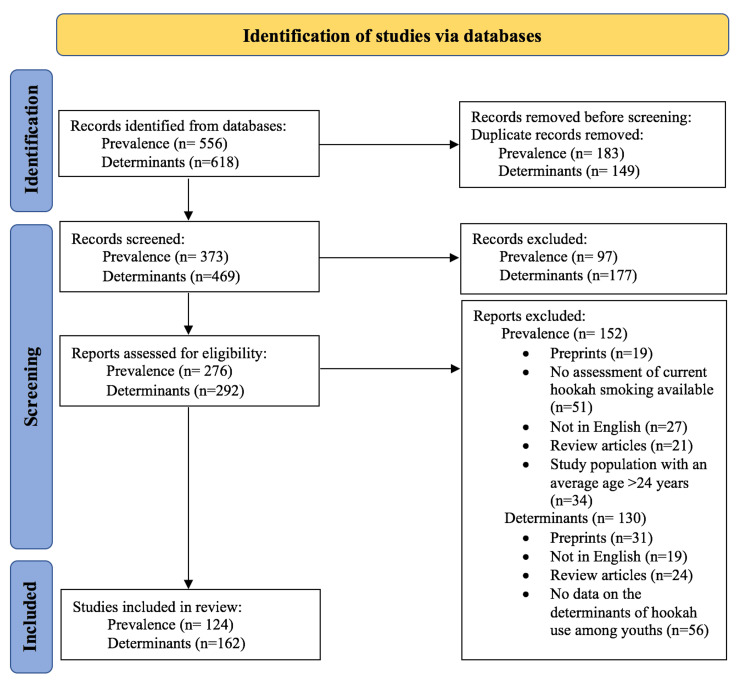
Screening process for the review of the prevalence and determinants of hookah smoking among youth Source: Page et al. 2021 [26]: The PRISMA 2020 statement: An updated guideline for reporting systematic reviews. BMJ. https://www.bmj.com/content/372/bmj.n71

The electronic search identified 556 and 618 records related to the prevalence and determinants (Figure 1), respectively, of hookah smoking. From these citations, 149 and 67 duplicates were excluded, respectively. The remaining abstracts were then screened from which 97 and 177 were excluded, respectively, for reasons such as conference abstracts and the lack of full texts.

Finally, the full-text articles were screened for eligibility based on the inclusion and exclusion criteria; articles were excluded for reasons such as published in a language other than English (27 and 19 articles, respectively), were preprints (19 and 31 articles, respectively), were review articles (21 and 24, respectively), included a study population with an average age exceeding 24 years (34 articles), did not examine the prevalence of hookah smoking (51 articles), and did not provide data on the determinants or predictors of hookah smoking (56 articles).

Data were extracted by two authors independently, T.J. and P.S., using Excel (version 15.37). Disagreements were discussed and resolved with D.M. The following data were extracted from the papers: author, aim, average age of participants, sample size, date of data collection, country, data collection tool, prevalence of hookah smoking, and age of hookah smoking initiation. The extracted results included the percentage of hookah smokers in the study population and the main predictors of hookah smoking.

Quality assessment and synthesis of findings

The methodological quality of all the included investigations was assessed independently by T.J. and P.S. through the use of the Newcastle-Ottawa Scale (NOS) for non-randomized studies [27]. This scale uses a "star" system that ranges from 0 (minimum) to 9 (maximum) to evaluate the quality of studies in three subscales: comparability of subjects; selection of participants; and the assessment of exposures/outcomes. Studies that scored seven to nine stars were deemed to be of high quality. Disagreements were discussed and resolved with D.M.

From the selected studies, findings related to the percentage of the study population that were current hookah smokers (smoked at least once within the past 30 days) were extracted, discussed in the manuscript, and displayed in a table format.

Findings related to the predictors/determinants of hookah smoking were extracted and presented under 12 different themes "Age," "Gender," "SES," "Geographic Region," "Other Addictive Habits," "Knowledge," "Relaxation, Fatigue Relief, Sensation, and Trend," "Flavor," "Social Environment," "Intention, Attitude, and Self-Efficacy," "Smoking Location," and "Policies/Ads." 

Results

The findings were grouped into two themes: the prevalence of hookah use and the main factors/determinants associated with this use among youth.

Hookah Use Assessment, Prevalence, and Age of Initiation

Assessment: We found that 124 studies met the inclusion criteria and assessed the prevalence of hookah use among youth in different countries across the world. The majority of these studies were cross-sectional [2-15,28-129], thus measuring its prevalence at one time point. The findings were displayed based on the country where they were conducted.

From the 124 investigations presented, most assessed hookah use via a self-administered questionnaire/survey [10,15,65,71,78,93] such as the Global Youth Tobacco survey [15,86,127,129]. Various studies also examined hookah use through an interview or interviewer-administered questionnaire [46,53,91,97,103,107,115].

Prevalence: Current hookah use in different countries was defined as smoking hookah at least once per month. Most investigations were conducted in the United States [100-123,130-132] and in different Middle Eastern countries such as Iran [2-5,40-57,133,134], Iraq [6,58,59], Lebanon [7,66,67], Palestine [76-78], Jordan [60-65,135], and Saudi Arabia [8,9,81-85]. Furthermore, various studies have been carried out in South Asia such as Pakistan [71-75] and India [36-38], in South Africa [10-12,87], and in different European countries including the United Kingdom [13,14,98,99] and Turkey [92-94].

In the US, prevalence mostly ranged from as low as 2% [114] to over 30% [113,130]. Sidani et al. [130] indicated in a prospective longitudinal study that the prevalence of hookah use increased from 36% to 49% between 2010 and 2011 in a group of university students in Florida, USA. Prevalence, however, was overall higher in Middle Eastern countries ranging from as low as 2% [49] to over 50% [4,65].

Age of hookah smoking initiation: Various research studies have denoted that youth initiate smoking during their early to mid-teen years [2-15]. Alzyoud et al. [62], for instance, indicated that the average age of hookah smoking initiation among a population of 1,000 Jordanian students was 11 (Table 1).

**Table 1:** Studies and summary of findings related to the prevalence of hookah use among youth _____, Data not available.

Author	Study Design	Sample Size	Aim	Location	Data Collection Tool	Date of Data Collection	Average Age/Age Range (Years)	Prevalence (Total and by Gender) %	Age of Hookah Smoking Initiation (Years)
Shayan et al. 2020 [28]	Cross-sectional	636	Assess the prevalence of hookah smoking and its association with sociodemographic characteristics in Herat University students.	Afghanistan	Questionnaire	2018	≤24	Total: 18.1% Males: 27% Females: 7.6%	________
Ahmed et al. 2020 [29]	Cross-sectional	340 university students	Examine hookah use and related behavior and normative beliefs among university students in Bangladesh.	Bangladesh	Questionnaire	2019	21.6	Total: 9.4%	_______
Martins et al. 2014 [30]	Cross-sectional	586 Med students	Assess the prevalence of hookah use and other forms of tobacco smoking among medical students, and their attitudes, beliefs, and knowledge.	Brazil	Questionnaire	2008-2013	22 (third-year Med student) 24 (sixth-year Med student)	Third-year Med student: Total: 47.5% Males: 53.4% Females: 40% Sixth year: Total: 46.4% Males: 51% Females: 40.7%	_______
Reveles et al. 2013 [31]	Cross-sectional	495	Assess the prevalence and factors associated with hookah use initiation among teenagers.	Brazil	Questionnaire	2011	≤19	Total: 19.7% Males: 10.4% Females: 19%	_______
Ngahane et al. 2023 [32]	Cross-sectional	1,008 students	Examine the prevalence, knowledge, and determinants of hookah use among university students.	Cameroon	Questionnaire	2020	22.9	Total: 26%	20
Minaker et al. 2015 [33]	Cross-sectional	27,404	Assess the prevalence, patterns of use, and perceptions of hookah smoking.	Canada	Youth Smoking Survey	2012-2013	≤17	Total: 5.4% Males: 6.6% Females: 4%	_______
Jiang et al. 2017 [34]	Cross-sectional	45,857	Examine the association of hookah smoking with cigarette smoking susceptibility and nicotine dependence among teenagers.	China	School-based survey	2012-2013	15.6	Total: 1.2% Males: 1.6% Females: 0.8%	_______
Lee et al. 2020 [35]	Cross-sectional	1288 university students	Assess the demographical and psycho-social factors and patterns of hookah use among university students.	China	Questionnaires	2018	22.4	Total: 23.8%	20.2
Hanewinkel et al. 2021 [136]	Cohort	Baseline: 3038; follow-up: 2752	Examine whether or not hookah smoking increases the risk of trying conventional and electronic cigarettes.	Germany	Questionnaires	2017-2018	14.87 at baseline; 14.85 at follow-up	Baseline: 12.5% Follow-up: 12.2%	______
Stamm-Balderjahn et al. 2012 [137]	Prospective case-control study with quasi-experimental design	760 school students in Berlin	Examine the impact of the educational intervention program called “Students in the Hospital”	Germany	Questionnaire	2007-2008	16	Total: 31.6%	_______
Anand et al. 2013 [36]	Cross-sectional	1,000	Assess the prevalence and main determinants of hookah use among high school students.	India	Questionnaire	2013	15.3	Total: 7.6%	15.7
Bali et al. 2015 [37]	Cross-sectional	215	Assess the characteristics, behavior, and perceptions associated with hookah use among the youth smokers	India	Questionnaire	2014	≤24	Total: 58.1%	17.3
Shaik et al. 2013 [38]	Cross-sectional	750	Assess the prevalence and main determinants of cigarette and hookah smoking in the age group of 17- 22 years	India	Questionnaire	2012	≤22	Total: 19%	18
Fauzi and Areesantichai 2022 [39]	Cross-sectional	1,318 high school students	Examine the factors associated with past 30-day hookah use among high school students in Jakarta, Indonesia.	Indonesia	Questionnaire	2015	≤19	Total: 5.20% Males: 8.4% Females: 3.3%	________
Abbasi-Ghahramanloo et al. 2016 [40]	Cross-sectional	1992 students	Assess the prevalence of hookah use and related factors in a group of medical sciences students.	Iran	Questionnaire	2012-2013	21.16	Total: 8.9% Males: 15.8% Females: 5.9%	________
Anbarlouei et al. 2018 [41]	Cross-sectional	1321 tenth-grade students	Assess the association between cigarette and hookah smoking, self-esteem, and communication skills among a group of high school students.	Iran	Questionnaire	2017	15.49	Total: 3.1% Males: 5.1% Females: 1.5%	______
Bashirian et al. 2016 [2]	Cross-sectional	601 high school male students	Assess the main determinants of hookah use among male high school students.	Iran	Questionnaire	2016	16.38	Total: 17.1%	13.39
Bashirian et al. 2018 [3]	Cross-sectional	730 high school male students	Assess the role of sociodemographic determinants of hookah use among male adolescents	Iran	Questionnaires	2017	16.41	Total: 26.3%	13.31
Bashirian et al. 2019 [133]	Randomized controlled trial	94 male adolescent students	Examine the impact of educational intervention based on Multi-Theory Model (MTM) to reduce hookah use among male adolescent students in Iran.	Iran	Questionnaire	2018	16.73	Intervention: 40.4%; control: 44.7%	13.6
Bashirian et al. 2020 [42]	Cross-sectional	1302	Assess the determinants of hookah use among adolescent females based on the Prototype-Willingness Model (PWM).	Iran	Questionnaire	2019	15.2	Total: 20.4%	13.65
Bashirian et al. 2021 [43]	Cross-sectional	1302	Examine the prevalence of hookah use and its associated risk factors among female teenagers.	Iran	Questionnaire	2019	15.22	20.4%	13.64
Bashirian et al. 2021 [134]	Randomized controlled trial	110 adolescent girls	Assess the impact of web-based educational program in the prevention of hookah use among girls	Iran	Questionnaire	2020	16.35 (experimental); 16.55 (control)	Experimental: 29.6%; control: 28.5%	Experimental: 12.86; control: 13
Fakhari et al. 2015 [44]	Cross-sectional	5197	Determine the prevalence and transition rates in hookah smoking statuses and the factors that predict these transitions among a group of Iranian high school students.	Iran	Questionnaire	2010-2011 6-month interval	15.7	Total: 6% Males: 10.4% Females: 1.4%	________
Ghafouri et al. 2011 [4]	Cross-sectional	296 first-year health sciences students	Examine the hookah smoking perceptions and practices of students in Iran and unravel the determinants related to the initiation and maintenance of hookah use among first-year health sciences university students.	Iran	Survey	2007	22	Total: 51% Males: 52% Females: 48%	16.9
Jeihooni et al. 2018 [45]	Cross-sectional	157 university students	Examine the knowledge and attitudes of university students regarding hookah use based on the theory of planned behavior (TPB).	Iran	Questionnaire	2015	23.14	Total: 32.3%	Not stated
Joveini et al. 2016 [46]	Cross-sectional	306 male students	Assess the determinants of hookah smoking among Sabzevar Azad University students.	Iran	Interview + Questionnaire	2014	22.4	Total: 46.9%	17.9
Karimy et al. 2013 [47]	Cross-sectional	380 Iranian male adolescents	Assess the association between refusal self-efficacy, self-esteem, smoking refusal skills, and hookah use	Iran	Questionnaire	2012	16.7	Total: 17.3%	_______
Karimy et al. 2013 [48]	Cross-sectional	400 male adolescents	Assess the attitudes, risk perception, and perceived vulnerability toward hookah use among male students	Iran	Questionnaire	2011-2012	16.7	Total: 18%	______
Kelishadi et al. 2016 [49]	Cross-sectional	13,486 children and adolescents	Examine the determinants of tobacco smoking and hookah use in a nationally representative sample of Iranian children and adolescents.	Iran	Questionnaire	2011-2012	12.47	Total: 1.8% Males: 2.49% Females: 1.14%	_______
Nabipour et al. 2016 [50]	Cross-sectional	682 students	Assess the association between hookah use and different dimensions of religiosity in a sample of students attending two major universities.	Iran	Questionnaire along with the Duke University Religion Index	_________	21.4	18.8%	_______
Rajabalipour et al. 2019 [51]	Cross-sectional	1,218 urban adolescents	Assess the risk factors and the prevalence of hookah use among adolescents based on the social cognitive theory (SCT).	Iran	Questionnaire	2017-2018	15.93	Total: 37.76% Males: 23.5% Females: 14.2%	_______
Rezaei et al. 2017 [52]	Cross-sectional	630 high school students	Examine the familial and social determinants of tobacco and hookah smoking among high school students	Iran	Questionnaire	2015	15.68	Total: 9.4% Males: 11.4% Females: 7.3%	_______
Roohafza et al. 2011 [53]	Cross-sectional	233 students	Examine the determinants of hookah smoking initiation and maintenance in Iranian university students.	Iran	Interviewer-administered questionnaire	2007	22	Total: 19.15% Males: 28.7% Females: 11.5%	_______
Roohafza et al. 2015 [54]	Cross-sectional	5,408	Better understand hookah and cigarette smoking based on perceived parental reaction and appeal.	Iran	Questionnaire	2010	14.37	Total: 11.5%	_______
Sabahy et al. 2011 [5]	Cross-sectional	1024 university students	Determine the prevalence of hookah use among Iranian university students and identify perceived factors for use.	Iran	Questionnaire	2010	20.6	Total: 18.7% Males: 28% Females: 9.5%	16.3
Sahebihagh et al. 2017 [55]	Cross-sectional	521 first-year students in QUMS	Examine the prevalence and determinants of substance abuse in first-year students of Qazvin University of Medical Sciences (QUMS).	Iran	Questionnaire	2014	19.6	4%	Not stated
Sadeghi et al. 2019 [56]	Cross-sectional	280	Assess the determinants for preventing hookah use in the youth, based on the protection motivation theory (PMT).	Iran	Questionnaire	2018	15.6	Total: 44.3%	________
Ziaei et al. 2016 [57]	Cross-sectional	1517 students	To assess the determinants of hookah use among 15-17-year-old high school students	Iran	Global Youth Tobacco Survey	2013-2014	16.1	Total: 9.7% Males: 13.5% Females: 6.2%	______
Al-Delaimy and Al-Ani 2021 [58]	Cross-sectional	847 male high school students	Assess the prevalence of hookah use among male high school students and investigate the factors associated with this use.	Iraq	Questionnaire based on the California Tobacco Surveys for tobacco use	2017-2019	≤18	Total: 46.1%	_______
Mousawi 2014 [6]	Cross-sectional	2298 students	Determine the prevalence and determinants of hookah smoking among university students	Iraq	Questionnaire	2005	21.2	Total: 10.5% Males: 21% Females: 0.6%	17.5
Othman et al. 2017 [59]	Cross-sectional	1061	Assess the prevalence of hookah smoking and its related factors.	Iraq	Questionnaire	2014-2015	22	Total: 28% Males: 49.4% Females: 9.4%	<18
Al-Sawalha et al. 2021 [60]	Cross-sectional	966	Assess the attitude, knowledge, and factors of knowledge of university students toward the harmful impacts of hookah use.	Jordan	Questionnaire	2019-2020	20	Total: 40%	______
Al-Sheyab et al. 2021 [61]	Cross-sectional	505 students	To examine the risk perceptions and behaviors of hookah smoking use among undergraduate medical and nursing students in Jordan.	Jordan	Questionnaire	2015	21.71	Total: 32.9% Males: 51.2% Females: 16%	______
Alzyoud et al. 2013 [62]	Cross-sectional	1,000 students	Examine the patterns and the determinants of hookah smoking among school-aged children	Jordan	Arabic Youth Tobacco Use Composite Measure (YTUCM) Questionnaire	2012	14.7	Total: 34% Males: 24% Females: 42%	11
Dar-Odeh et al. 2010 [63]	Cross-sectional	1454 students	Assess the hookah use prevalence, patterns, and beliefs related to adverse health among students in three public Jordanian universities	Jordan	Questionnaire	2008	≤24	Total: 36.8% Males: 61.9% Females: 10.7%	_______
Khabour et al. 2012 [64]	Cross-sectional	1845	Assess the prevalence of hookah use and cigarette smoking among university students and their demographic and environmental determinants	Jordan	Questionnaire	2010	21.4	Total: 30% Males: 59% Females: 13%	<21
McKelvey et al. 2014 [135]	Longitudinal cohort	1781	Examine the determinants of hookah smoking guided by the attitude-social influence-self Efficacy (ASE) theory	Jordan	Questionnaire	2008-2011	13	Initiation over 3 years: Total: 36.8%; Males: 39.2%; Females: 28.3%	______
Obeidat et al. 2014 [65]	Cross-sectional	547 students	Assess the prevalence, social acceptance, and awareness of hookah use among dental university students.	Jordan	Questionnaire	2013	≤24	Total: 54.8% Males: 36.6% Females: 88.6%	18.1
El-Roueiheb et al. 2008 [7]	Cross-sectional	2,443	Examine the tobacco smoking practices (hookah and/or cigar) among teenagers from public and private schools	Lebanon	Questionnaire	2003-2004	15	Total: 29.6% Males: 26.3% Females: 25%	12.1
Jawad et al. 2015 [66]	Cross-sectional	1128 sixth- and seventh-grade students	Assess the prevalence, patterns, and determinants of hookah use among students.	Lebanon	126-item tobacco questionnaire	2011-2012	12.3	Total: 22.1% Males: 22% Females: 22%	_______
Jradi et al. 2013 [67]	Cross-sectional	191	Assess the prevalence of cigarettes and hookah smoking and examine the knowledge and behavior factors among sixth-year medical students	Lebanon	Questionnaire	2009-2010	23.6	Total: 29.5% Males: 35.8% Females: 21.2%	18
Al-Naggar et al. 2012 [68]	Cross-sectional	300 medical students	Assess the prevalence of hookah use and associated factors	Malaysia	Questionnaire	2011-2012	22.5	Total: 20%	______
Redhwan et al. 2011 [69]	Cross-sectional	200 university students	Assess the use and determinants of hookah use.	Malaysia	Questionnaire	2010-2011	≤24	Total: 30%	______
Otakhoigbogie et al. 2022 [70]	Cross-sectional	546 university students	Examine the knowledge and sociodemographic determinants of hookah use among university students	Nigeria	Questionnaire	2019	20.55	Total 24.7% Males: 25.1% Females: 24.4%	_______
Habibullah et al. 2013 [71]	Cross-sectional	7145	Examine the prevalence of hookah use, its trends and determinants in college, university, and school students	Pakistan	Survey	2010-2011	21	Total: 19.7% Male 29.8% Female 10.4%	<18
Jaffri et al. 2012 [72]	Cross-sectional	422	Assess the frequency of hookah use among university students and examine their practices, knowledge, and attitude.	Pakistan	Questionnaire	2009	21.6	Total: 22.1%	_______
Khan et al. 2010 [73]	Cross-sectional	1204	Assess the impact of age and gender on knowledge, attitude, and practice of hookah use among medical and dental students.	Pakistan	Questionnaire	2007	≤24	Total: 22.8% Males: 41% Females: 16.9%	________
Masood and Sohail 2013 [74]	Cross-sectional	1,000 students	Assess the perceptions of hookah smoking among university students.	Pakistan	Questionnaire	2011-2012	≤24	Total: 49.92% Males: 59.22% Females: 22%	_______
Shuja et al. 2018 [75]	Cross-sectional	342 students	Assess the perception of health professional students regarding hookah use and their awareness of its harmful effects on oral health.	Pakistan	Questionnaire	2015-2016	21.36	Total: 10%	_______
Musmar 2012 [76]	Cross-sectional	954	Examine the prevalence and attitude of university students toward hookah smoking.	Palestine	Questionnaire adopted from the Global Health Professionals Survey and the Global Youth Tobacco Survey	2009-2010	20.5	Total: 34.7% Males: 52.7% Females: 16.5%	_____
Nazzal et al. 2020 [77]	Cross-sectional	810 university students	Examine the prevalence and determinants of hookah smoking among students at a major Palestinian university.	Palestine	Questionnaire	2014	≤24	Total: 22.8% Males: 35.4% Females: 11.8%	17
Tucktuck et al. 2017 [78]	Cross-sectional	1891	Assess the prevalence of hookah and cigarette smoking and identify the associated factors among a sample of university students.	Palestine	Questionnaire based on the Global Adults Tobacco Survey (GATS)	2014-2015	20.1	Total: 24.4% Males: 36.4% Females: 12.9%	17
Al-Jayyousi et al. 2022 [79]	Cross-sectional	199	Assess the prevalence and the individual, sociocultural, and environmental determinants of hookah use among university students.	Qatar	Questionnaire	2020	19	Total: 40%	_______
Omotehinwa et al. 2018 [80]	Cross-sectional	427 university students	Determine the prevalence and main determinants of hookah use among students in a university in Kigali Rwanda.	Rwanda	Questionnaire	2016-2017	23.8	20.8%	_______
Almogbel et al. 2021 [81]	Cross-sectional	928 twenty-one to twenty-three-year olds	Assess the determinants of hookah use among university students.	Saudi Arabia	Questionnaire	2018 and 2019	21-23	Total: 37.4%	_______
Al Mohamed and Amin 2010 [82]	Cross-sectional	1,382	Assess the prevalence of different forms of smoking and its main determinants.	Saudi Arabia	Questionnaire based on the Global Youth Tobacco Survey plus the modified Fagerström Test for Nicotine Dependence	2006-2007	20.9	Total: 14.6%	_______
Alzohairy 2012 [8]	Cross-sectional	500 male students	Assess the prevalence and beliefs of hookah and cigarette smoking among male students.	Saudi Arabia	Questionnaire	2011	21.41	Total: 40%	16.43
Amin et al. 2012 [83]	Cross-sectional	1,652 students	Assess hookah use prevalence, knowledge of associated health effects, attitude, and psychosocial determinants among secondary school adolescents.	Saudi Arabia	Arabic version of the Global Youth Tobacco Survey	2008	17.5	Total: 8.1% Males: 8.1% Females: 0.0006%	______
Awan et al. 2016 [84]	Cross-sectional	535	Examine the prevalence of hookah use among healthcare university students and their attitudes and practices.	Saudi Arabia	Questionnaire	2015	24.0	23%	18.1
Koura et al. 2011 [9]	Cross-sectional	1,020	Assess the prevalence and the smoking patterns among female non-medical college students in Dammam, Saudi Arabia.	Saudi Arabia	Modified WHO Global Youth Tobacco Survey questionnaire	2005	20.4	Total: 3.72%	16
Taha et al. 2010 [85]	Cross-sectional	371 male students	Assess the use and determinants of hookah use among male students of three colleges.	Saudi Arabia	Questionnaire	2008	≤24	8.6%	16-18
Othman et al. 2019 [86]	Cross-sectional	3,387 students	Assess the prevalence of hookah smoking and its determinants among adolescents.	Sudan	Questionnaire based on Arabic version of the Global Youth Tobacco Survey	__________	≤17	Total: 13.4% Male 16.8% Female 10.9%	________
Daniels and Roman 2013 [10]	Cross-sectional	389 university students	Examine the beliefs, and associated behaviors, related to the health risk of hookah use.	South Africa	Questionnaire constructed from the College Health Behaviour Survey	2010-2011	22.2	40%	15.7
Naicker et al. 2020 [11]	Cross-sectional	579 students	Examine hookah use and determinants among eighth and 12th graders.	South Africa	Questionnaire	________	Grade 8: 13.9 Grade 12: 18.1	Grade 8: 10.9% Grade 12: 37.1%	Grade 8: 8 Grade 12: 12
Kruger et al. 2016 [12]	Cross-sectional	4,578	Examine prevalence of hookah and cigarette smoking and associated determinants.	South Africa	Questionnaire	2013	≤24	Total: 9.9%	16.17
Van der Merwe et al. 2013 [87]	Cross-sectional	228	Assess the knowledge, attitudes, and practices of hookah use among health sciences students.	South Africa	Questionnaire	2013	21.4	Total: 18%	________
Ramji et al. 2015 [88]	Cross-sectional	106 adolescents who attended an urban high school in northern Sweden	Determine the prevalence and determinants of hookah use in a sample of adolescents in northern Sweden.	Sweden	Questionnaire	2013	≤19	Total: 25%	________
Albisser et al. 2013 [89]	Cross-sectional	201	Unravel the sociodemographic characteristics of hookah users in Switzerland and examine concurrent cigarette and cannabis smoking habits.	Switzerland	Questionnaire	_________	21	Total: 30%	_______
Alolabi et al. 2020 [90]	Cross-sectional	622 university students	To assess the prevalence of cigarette and hookah smoking among university students, and examine the addictive behavior.	Syria	Survey	2019	21.3	Total: 18%	_______
Maziak et al. 2004 [91]	Cross-sectional	587 students	Examine the beliefs and attitudes related to hookah smoking likely to be associated with its use and increased popularity among the youths	Syria	Interviewer-administered questionnaire	2003	21.8	Total: 14.7% Males: 25.5% Females: 4.9%	20
Erbaydar et al. 2010 [92]	Cross-sectional	460	Assess the knowledge, smoking patterns, and the perceptions of hookah use in Turkey.	Turkey	Questionnaire	2004-2005	22.5	Total: 40.2%	_______
Karaman et al. 2022 [93]	Cross-sectional	411 university students	Examine the frequency and patterns of hookah use among university students and assess their perception on its health risks.	Turkey	Questionnaire	________	22.15	Total: 38.4% Males: 29.1% Females: 70.9%	17
Poyrazoglu et al. 2010 [94]	Cross-sectional	645 students	Examine the prevalence of hookah use among students and its main sociodemographic determinants.	Turkey	Questionnaire	2008-2009	20.3	Total: 32.7% Males: 41.6% Females: 20.2%	17.4
Al-Rawi et al. 2018 [95]	Cross-sectional	397 students	Examine hookah smoking among dental school students	UAE	Survey	2016	≤24	Total: 14.11% Males: 43.3% Females: 8.2%	________
Saravanan et al. 2019 [96]	Cross-sectional	633 students	Assess the prevalence of ever hookah, current hookah, and hookah dependency use among university students in the University of Sharjah (UOS).	UAE	Knowledge and Belief scale, Modified Reason for Smoking Scale, and Fagerstrom Test for Nicotine Dependence (FTND)	2017-2018	20.9	Total: 38.9%	________
Aanyu et al. 2019 [97]	Cross-sectional	663	Examine hookah use and its main determinants to bolster public health interventions.	Uganda	Interviewer-administered semi-structured questionnaire	2014	24	Total: 36.4%	22.8
Jawad et al. 2013 [13]	Cross-sectional	2,399 students	Assess the prevalence and determinants of hookah use and cigarette smoking among students attending secondary schools in an underserved and ethnically diverse part of inner London.	UK	Questionnaire	2011-2012	14.5	Total: 7.6% Males: 7.1% Females: 8.1%	13.5
Jawad and Power 2016 [14]	Cross-sectional	2,098	Assess the prevalence of hookah use among secondary school students.	UK	Survey	2014	13.7	Total: 5%	14.2
Jawad et al. 2016 [98]	Cross-sectional	2,217	Assess the prevalence and patterns of hookah use and examine the tobacco control policy.	UK	Survey	2013-2014	23.4	Total: 14.4% Males: 12.2% Females: 8.7%	_______
Jawad et al. 2018 [99]	Cross-sectional	7,954	Examine hookah use prevalence and correlate among young people in Great Britain	UK	Survey	2013-2016	≤18	Total: 1.7%	_______
Agaku et al. 2018 [100]	Cross-sectional	20, 675 sixth to 12th graders	Assess the three social dimensions of youth hookah use: frequency, places smoked, and descriptive social norms.	USA	2016 National Youth Tobacco Survey	2016	≤18	Total: 26.3%	
Barnett et al. 2014 [101]	Cross-sectional	Florida high school students	Examine the prevalence of hookah use among Florida high school students over time.	USA	Florida Youth Tobacco Survey	2009-2012	≤18	Total: 7.7% in 2009 and 2012 Males: 9.1% in 2009 and 8.2% in 2012 Females: 6.4% in 2009 and 7% in 2012	_______
Barnett et al. 2013 [102]	Cross-sectional	852 students	Assess the associations between positive and negative attitudes and hookah use among college students.	USA	Survey	2010	20.6	Total: 14%	_______
Barnett et al. 2013 [103]	Cross-sectional	1,203 university students	Examine hookah use and unravel the associations with cigarette smoking and demographic factors.	USA	Computer-aided survey	2012	≤24	Total: 9.9% Males: 11.9% Females: 8.2%	_______
Barnett et al. 2017 [104]	Cross-sectional	Florida high school students	Examine the trends in the prevalence of lifetime hookah use and current hookah use among high school students (grades 9–12).	USA	Florida Youth Tobacco Survey data	2011-2014	≤18	Total: 8% Males: 9.3% Females: 6.7%	________
Braun et al. 2012 [105]	Cross-sectional	445	Examine the perceptions, knowledge, and beliefs of hookah use among university students.	USA	Survey	_________	23.1	Total: 6%	________
Creamer et al. 2016 [106]	Cross-sectional	5,451 young adults	Examine college students’ use and awareness of tobacco and nicotine content in hookah.	USA	Survey	2014-2015	20.49	Total: 16.8%	________
Doran et al. 2015 [107]	Secondary data analysis of a longitudinal study	256 college students	Examine whether or not added nicotine exposure from hookah smoking may increase the uptake of cigarettes.	USA	Two in-person interviews	_________	19.8	Total: 34%	________
Fevrier et al. 2018 [108]	Cross-sectional	403 college students	Examine the possible patterns/differences in college students’ hookah use.	USA	Survey	________	22.2	Total: 8.8%	________
Heinz et al. 2013 [109]	Cross-sectional	143 undergraduate students	Assess the patterns, contexts, determinants, social norms, and health perceptions of hookah use.	USA	Questionnaire	2013	19.26	Total: 22%	17-18
Huang et al. 2017 [110]	Cross-sectional	4,092	Assess the changes in the prevalence of hookah use and its main determinants from 2011 to 2013 among North Carolina high school students.	USA	NC Youth Tobacco Survey	2011-2013	≤18	Total: 3.6% in 2011 Total: 6.1% in 2013	________
Jordan and Delnevo 2010 [111]	Cross-sectional	3,010 high school students	Assess the prevalence factors associated with hookah smoking among high school students.	USA	Questionnaire	2008	≤18	Total: 9.7% Males: 10.47% Females: 8.79%	________
Kassem et al. 2015 [112]	Cross-sectional	1,332 undergraduate students	Assess hookah tobacco use, hookah lounge attendance, and the factors and impediments related to hookah lounge attendance.	USA	Survey	2007	21.2	Total: 72.8%	18.3
Krenik-Matejcek et al. 2017 [113]	Cross-sectional	200 students	Examine college students’ behaviors, attitudes, and knowledge regarding hookah use.	USA	Survey	Published 2017	21	Total: 32%	17.9
Leavens et al. 2018 [114]	Cross-sectional	894 college students	Assess the perceived and actual descriptive and injunctive norms of hookah use among a college student sample.	USA	Questionnaire	2016	19.67	Total: 2.2% Males: 3.3% Females: 1.7%	________
Lee et al. 2014 [115]	Cross-sectional qualitative	San Diego (N = 2,243), Tulsa (N = 2,095), Oklahoma city (N = 2,200), Albuquerque (N = 1,044), and Las Cruces (894)	Assess differences in tobacco-related attitudes, hookah, and cigarette use among college and non-college young adults.	USA	Qualitative interviews with key informants, patrons in focus groups, and bar owners	2009-2011	≤24	Total: 21.3%	________
Noonan et al. 2011 [116]	Cross-sectional	1,000 undergraduate students	Assess the association between behavioral beliefs, attitudes, normative beliefs, and subjective norms and hookah use intention in college students.	USA	Survey	2007	19.9	Total: 13.5%	________
Nuzzo et al. 2013 [117]	Cross-sectional	852	Examine the knowledge of hookah use’s harmful effects compared to cigarette smoking and assess the associations between this knowledge and hookah smoking outcomes.	USA	Survey	2010-2011	20.6	14%	________
Primack et al. 2013 [118]	Cross-sectional	105,012	Assess the prevalence of hookah use in a large diverse sample of US university students and examine the individual and institution-related determinants.	USA	Survey	2008-2009	22.1	Total: 8.4%	________
Rahman et al. 2012 [119]	Cross-sectional	478 undergraduate and graduate students	Assess the prevalence of hookah use and its social and behavioral determinants.	USA	Survey	2011-2012	≤24	Total: 16.3% Men: 22.2% Women: 11.5%	________
Rezk-Hanna et al. 2014 [120]	Cross-sectional	91	Utilize the health belief model to assess the perceptions, attitudes, beliefs, and preferences of hookah use and to unravel the main determinants that may impact heavy versus light hookah use among young adults.	USA	Survey	________	23.6	Total: 20.9%	19
Sidani et al. 2014 [130]	Prospective longitudinal cohort	Baseline: n = 852 Follow-up: n = 569	Assess the associations between knowledge, attitudes, and normative beliefs and initiation of hookah use among university students	USA	Survey	_________	≤24	Baseline: 36% Follow-up: 49%	________
Sidani et al. 2019 [121]	Cross-sectional	3,236	Assess the attitudes, normative beliefs, certain sociodemographic factors, and current hookah use among young adults not attending college and compare them to young adults in college.	USA	Survey	2013	24	3% not in college, 7% in college	________
Smith et al. 2011 [122]	Cross-sectional	689 students	Assess hookah use initiation, prevalence, cessation, and its psychosocial determinants among high school students.	USA	Survey	2010	17.1	Total: 10.9%	15.8
Sterling and Mermelstein 2011 [131]	Longitudinal	951 adolescents	Assess hookah use and determinants among a sample of teenagers who have ever smoked and may be at high risk for smoking hookah.	USA	Questionnaire	_________	17.6	Total prevalence: 17% Males: 19% Females: 15.2%	________
Sutfin et al. 2011 [123]	Cross-sectional	3,770 college students from eight universities	Determine the prevalence of hookah use among a large, multi-institution sample of college students and unravel its main determinants.	USA	Survey	2008	≤24	Total: 17.4% Males: 23.8% Females: 13.7%	17.9
Villanti et al. 2015 [132]	Cohort	1,150	Assess the determinants of hookah use and hookah initiation.	USA	Questionnaire	2013-2014	≤24	Total: 4%	______
Nasser et al. 2018 [124]	Cross-sectional	380	Assess the prevalence, attitudes, and determinants of smoking among college students in the rural area of Hajja, Yemen.	Yemen	Questionnaire	2016	21.43	Total: 5% Males: 0% Females: 13%	________
Nasser and Zhang 2019 [125]	Cross-sectional	420	Assess the smoking behavior and smoking-related knowledge among students.	Yemen	Global Youth Tobacco Survey and a Global Health Professional Survey	2017	21.93	Total: 9.3% Males: 1.9% Females: 21.1%	________
Hawash et al. 2022 [126]	Cross-sectional	2,030 selected university students	Assess the university students’ perception of hookah smoking addictions and the main determinants behind the rise in the prevalence of hookah smoking.	Palestine, Jordan, and Turkey	Questionnaire	_________	≤24	31.8%	________
Jawad et al. 2016 [127]	Cross-sectional	76,185	Examine the prevalence and determinants of hookah use among young people worldwide.	Various countries around the world	Global Youth Tobacco Survey	_________	≤24	Lebanon (36.9%), West Bank (32.7%), Parts of Eastern Europe (Latvia 22.7%, the Czech Republic 22.1%, Estonia 21.9%)	________
Ma et al. 2022 [15]	Cross-sectional	335,062 adolescents	Examine the prevalence of, and trends in, hookah use and its correlates among teenagers in 73 countries/territories	73 countries/territories	Global Youth Tobacco Survey	2010-2019	14.6	Total: 6.9% Males: 8.5% Females: 5.3%	<13
Salloum et al. 2019 [128]	Cross-sectional	Egypt (n = 728), Jordan (n = 790), and Palestine (n = 722)	Assess hookah smoking patterns, location of smoking, and prices paid among university students in the Eastern Mediterranean Region.	Egypt, Jordan, and Palestine	Survey	2018	21.6	Total: 60.7%, 67.7%, and 63.1% of students from Egypt, Jordan, and Palestine, respectively	17.3
Veeranki et al. 2015 [129]	Cross-sectional	30,711 never-smoking adolescents	Examine the association of hookah use with susceptibility to cigarette smoking among never-smoking youth.	17 Arab nations	Global Youth Tobacco Survey	2002-2011	14	Total: 5.2% Males: 6.3% Females: 4.2%	_______

Determinants of Hookah Smoking

We identified 162 articles that examined the factors associated with hookah use among youth including demographics, geographic region, knowledge, social environment, correlation with other addictive habits, sensation seeking, intention, policies, and ads. The full list of references for each determinant is displayed in Appendix Table 2.

Age: The majority of the investigations have indicated a positive association between age and hookah use among youth [77,80,81,97,99,126,138]. According to Alzyoud et al. [62], with every one-year increase in age, the odds of being a 30-day hookah smoker increased by 1.6 times. This suggests that youth are more likely to report being a current hookah smoker and/or engage in hookah smoking as they age and might have increased exposure to more correlated risk factors [5,62]. The older the youth, the more likely to become independent, employed, and distant from their parents, and the more likely they are to smoke [81,126].

Gender: Research consistently has shown that males are more likely to be hookah smokers than females [14,50,118,120,125,139]. A cross-sectional study was conducted by Khabour et al. [64] in Jordan on 1845 university students and showed that females had significantly lower odds of being current hookah users (OR = 0.12) compared to males [64]. The differences could be partly explained by the less tolerance toward female smoking behavior and the lack of access to places available for females’ hookah use in various regions such as Saudi Arabia [83], Jordan [64], and Yemen [125]. Nevertheless, the gender gap in hookah smoking prevalence has been decreasing especially in Middle Eastern Regions since hookah use is considered a socially acceptable practice [140].

Socioeconomic status (SES): SES has been shown to be positively associated with hookah smoking behavior among young people [29,33,98,124,125,141]. A cross-sectional survey by Palamar et al. [142] that examined 5540 high school seniors in ∼130 public and private schools throughout 48 US states showed that the student's weekly income from a job of >$50/week significantly increased the odds for hookah use compared to <$50/week. This is not surprising since wealthier students can afford to purchase a hookah device and hookah tobacco and can regularly visit the hookah lounges. Various studies have also indicated that parental education level is also positively associated with hookah smoking among youth [6,28,85,127,142,143]. This supports the hypothesis that hookah use is considered a social activity that occurs frequently among individuals of higher SES.

Geographic region: Hookah smoking is most prevalent in various geographic regions such as Middle Eastern countries including Iran [2-5,40-57,133,134], Jordan [60-65,135], Saudi Arabia [8,9,81-85], Palestine [76-78], Lebanon [7,66,67], Iraq [6,58,59] along with South Asia including India [36-38], Pakistan [71-75], and Bangladesh [138]. Research also has indicated that even in Western countries such as the UK [13,14,98] and the United States [100-123,130-132], hookah smoking was most prevalent among Middle Eastern and South Asian ethnicities. Hookah smoking is a common practice among these ethnicities since it is part of their culture and as a means to relax and have a social activity to share with others.

Other addictive habits/substance abuse: There is strong and consistent evidence suggesting a positive association between cigarette smoking and hookah use among youth [8,39,77,88,110]. For instance, Rahman et al. [119], in a cross-sectional study involving 478 undergraduate and graduate students from Florida, USA, confirmed this finding and showed that smokers had much higher odds of smoking hookah than non-smokers with an odds ratio (OR) of 4.52. Cigarette smokers might perceive hookah to be a safer alternative for smoking and thus might be more susceptible to using hookah than non-cigarette smokers. Moreover, several studies have also indicated that alcohol [6,12,32,144,145], marijuana [89,109,132,146], and even illicit drug use [123,142] are positively related to hookah smoking since the involvement in one risky behavior is associated with engagement in other risky behaviors [147,148].

Knowledge: Literature strongly indicates that knowledge and beliefs were two of the strongest predictors of hookah smoking. Investigations have consistently shown that youth perceived hookah smoking to be less harmful than cigarettes [37,149-151] and that it is either not addictive or less addictive than other forms of smoking [10,83,152-156]. Common assumptions regarding hookah smoking include the ability of water in the waterpipe to absorb all the “impurities” in tobacco [153] and the purification of harmful substances through water filtration [83,149]. Studies also have suggested that youth believed hookah contained less nicotine [28,68], is less harmful than cigarettes in terms of oral health [65], and is less likely to cause cardiovascular diseases or dental problems [79]. 

There is either a lack or deficit of knowledge of the harmful consequences of hookah use [32,72,73,84,88,113,120,130]. Krenik-Matejcek et al. [113] confirmed this in their cross-sectional study conducted on 200 students in the USA and showed that the overall mean knowledge score regarding the health impacts of hookah use was only 4.4 questions correct out of 10 [113]. Al-Naggar and Bobryshev [68] indicated in a study conducted in Malaysia on 300 medical students that many participants believed that hookah contains no carbon monoxide or nicotine and does not contribute to lung cancer, cardiovascular diseases, or dental issues. According to Shuja et al. [75] and Krenik-Matejcek et al. [113], over 30% of the participants were unaware that hookah use was the reason for stained teeth, dental caries, bad taste, and halitosis [75] and that it can contribute to oral cancer [113].

Relaxation, fatigue relief, sensation, and trend:* *It was portrayed by various investigations that youth use hookah to smoke as a means for relaxing, relieving fatigue [10,38,74,93,105,113], managing stress and anxiety [9,38,46,74,156], and seeking pleasure and sensation [124,136,153,156]. Sharma et al. [153] performed in-depth, in-person, semi-structured qualitative interviews to assess the determinants of hookah use among 1517 fifteen to seventeen-year-old high school students and showed that one of the main reasons why youth smoke hookah had to do with the physiological effects commonly referred to as “buzz.” Literature also suggests that youth might be smoking hookah for fun, pleasure-seeking, and offering an alluring way to spend leisure time with others. According to Ghafouri et al. [4], 75.5% of hookah users indicated that fun was one of the main factors for continuing to smoke hookah. Moreover, studies have denoted that hookah’s growing popularity is one of the key predictors of its prevalence among youth and that they viewed hookah as stylish, cool, and trendy [38,74,91,111,155,157]. 

Flavor: Flavor is one of the main reasons hookah smoking is common among youth [54,88,158,159]. Youths smoke hookah due to the desirable taste and pleasant smell. Many enjoy the fruity flavors of hookah [71,97,149,160]. Aanyu et al. [97], for instance, denoted that 97.4% of the study population in Uganda smoked flavored and sweetened tobacco [97].

Social environment:* *The social environment was the strongest predictor of hookah smoking [73,80,149,150]. Youth indicated that they smoke hookah because of having a friend or family member who smokes hookah and/or as a means to spend leisure time or to socialize with friends [8,9,11,39,48,60,105]; many considered it to be socially acceptable [2,95,156,159]. The majority of youth have also stated that they share the same hookah during the smoking sessions [59,97,161]. Othman et al. [86] conducted a cross-sectional study on 3387 students in Sudan and denoted that having friends who smoke hookah was associated with higher odds of being a hookah smoker (OR 2.39). Jeihooni et al. [45] noted that youth continued using hookah due to the concern that its cessation might contribute to the loss of contact with friends [45]. 

Aside from friends, one of the main predictors of hookah smoking was having a family member who smokes hookah. Thus, youth are more likely to smoke if a parent, sibling, or a relative already smokes hookah. Al-Rawi et al. [95] suggested that having a smoker sibling led to an increase in the odds of being a smoker by 4.52 times. It is a common aspect of the culture in many regions around the world to smoke hookah among family and friends since it is more acceptable among parents than other forms of tobacco smoking [54,65,160,162]. Obeidat et al. [65] denoted that over 30% and 60% of male and female hookah smokers, respectively, stated that their parents were indifferent toward their use of hookah.

Intention, attitude, and self-efficacy:Studies overall have revealed that the majority of youth did not have the intention of quitting hookah smoking [57,87,98,116]. Bali et al. [37], however, indicated that around 61% of participants had made an attempt to quit smoking but restarted. According to Wong et al. [157], hookah users who have been smoking for less than 12 months were significantly more likely to have the intention to quit smoking compared to hookah users who have been smoking for 12 months or more.

Research also strongly indicated that having a positive attitude was positively associated with hookah smoking [48,72,102,108]. A positive attitude toward hookah use could be explained by the perception that hookah smoking is not as harmful as other smoking means and by the acceptance of hookah smoking by the family and culture. In addition to intention and attitude, low hookah refusal self-efficacy [47,51,135,143,163] and curiosity [9,74,80,84,92,98,150,164] have been shown to be the main predictors of hookah smoking.

Accessibility and smoking location:Cafes [37,58,59,65,71,152] and hookah lounges [36,73,112,122,123] were two of the most common locations for hookah smoking. Since hookah smoking is a social activity, such locations are common places where youth meet to socialize and spend leisure time. Studies also showed that other common places for hookah smoking included a friend’s house [10,14,87,100] or home [9,10,43,48,65,100,159]. The acceptability of hookah use by families paves the way for youth to smoke hookah at home.

Accessibility to hookah is, thus, one of the main determinants of hookah use among youth [13,58,88,123,150]. For instance, the presence of a hookah cafe near youth residences or schools was significantly associated with hookah smoking [13,58,123]. Kassem et al. [112] indicated that the odds of ever visiting a hookah lounge were 2.1 times higher among youth whose university campus was <5 miles away from a hookah lounge compared to those ≥5 miles away.

Policies/Ads: Very few studies examined the influence of laws/policies on hookah smoking among youth [86,98,143,160]. Azodi et al. [160] indicated that the “lack of enforcement of prohibiting laws about the use of hookah in public” was a significant cause for the increase in the prevalence of hookah smoking among youth. Furthermore, a few studies have suggested that exposure to advertisements was a predictor of hookah smoking [15,58,66,82,110,165]. However, Salloum et al. [166], Jawad et al. [98], and Jaber et al. [143] denoted that seeing warning labels and health warnings might have been protective against hookah smoking. Yet even with the rise in its popularity, there is a lack of media attention that is addressing this issue [165].

Discussion

Hookah smoking is mostly prevalent among youth from the Middle Eastern region [8,64,65,81,82,84,135], South Asia [36-38,71-75], USA [100-123,130-132], and various European countries [92-94,99]. Research on the predictors of hookah smoking suggests that age [76,77,80,99,138], the male gender [11,29,61,88,89,167], SES [39,168], Middle Eastern or South Asian ethnicity [9,58,83-85], cigarette smoking [34,39,44,67], other forms of substance use such as alcohol drinking [6,12,80,142,144,145], knowledge and beliefs on hookah’s harm and addiction [48,74,83,154,169], stress management [9,38,46,74,156], sensation seeking [2,124,156], hookah flavor [71,88,92,97,149,159], having friends and/or family members who smoke hookah [55,92,150,156,167], social acceptability [2,150,156,159,165], intention [57,87,98,116], positive attitude [48,80,102,108,116], low hookah refusal self-efficacy [47,51,135,143,163], curiosity [74,80,92,150], accessibility to cafes and hookah lounges [13,58,123], and lack of enforcement of prohibiting laws [160,165] were positively associated with hookah smoking among youth. Common assumptions regarding hookah smoking included that water in the waterpipe absorbed all the “impurities” in the tobacco [153], that harmful substances are purified through water filtration [83,149], that it contains less nicotine than other forms of smoking [28,68], and that it is less harmful than cigarettes in terms of oral [65] and cardiovascular [79] health.

Previous systematic/literature reviews, while not focused on youth, have also confirmed that a large spectrum of individual and social factors can predict hookah smoking including positive viewpoints toward smoking hookah, wrong beliefs about its harmfulness and addictiveness, sensation and relaxation seeking, ease of accessibility, wrong cultural habits, and a way to socialize and connect with others [18-21]. Moreover, parallel to our findings, Jawad et al. [20] indicated that hookah smoking was most prevalent in the Eastern Mediterranean. In this region, people smoke hookah as part of their tradition, for relaxation, and for connecting with others.

Targeting the high prevalence and increasing popularity of hookah smoking among youth is imperative since it is highly detrimental to health and can place smokers and those around them at serious health risks [16]. Contrary to what many individuals believe, smoking hookah, just like smoking cigarettes, is associated with various acute and chronic health effects since there are at least 82 toxic chemicals and carcinogens in hookah smoke [16]. Short-term use contributes to various dire health impacts including increased risk of infections, blood pressure, heart rate, and carbon monoxide intoxication and reduced pulmonary function. Long-term use impairs pulmonary function and leads to elevated risk of a variety of chronic diseases such as chronic obstructive pulmonary disease, cardiovascular disease, and various cancers including lung, esophageal, bladder, gastric, and oral cancers [16].

Youth perceive themselves as invincible, believe that their health is not easily compromised, and are highly adventurous and not very health conscious. It is thus critical to provide education to increase knowledge regarding the harmful health repercussions of hookah smoking, its addictiveness, and its chemical and carcinogen contents. Previous studies have suggested that educational interventions on hookah use among youth can successfully decrease the prevalence of hookah smoking [137,170] by enhancing self-efficacy [133,171], attitude, and intention to quit [134,170-172]. Comprehending and targeting the family's attitude, perception, and knowledge of hookah smoking is highly imperative [91] since they play a key role in influencing youth, introducing hookah smoking, and controlling their relationships and leisure time. Evidence suggests that family‐based interventions can have a positive impact on preventing youth from initiating smoking [173]. Furthermore, since youth are influenced by their peers, peer-to-peer prevention initiatives are recommended during which a few socially influential student leaders are selected and trained in communication skills and techniques, to effectively communicate with their peers about the harmful and addictive repercussions of hookah smoking [174]. Public health authorities should establish educational programs that reach youth in schools and universities. Teachers and faculty at schools should update their curriculum and include tobacco and hookah prevention programs to educate youth on the negative health consequences and addictive qualities of hookah smoking and discuss available prevention and treatment options for tobacco addiction [4]. University administrators should strongly advocate for reducing the prevalence of hookah use on and off their campuses [4]. Since hookah bars, lounges, and cafes are considered important sources of exposure to hookah smoking for youth, restricting access to such places should be considered [131,175]. A review of smoke-free laws indicated that many of the major US cities that prohibited cigarette smoking in bars had exemptions for hookah smoking [176]. Thus, strict regulations should be set for the access of hookah smoking for youth and for the production, distribution, marketing, and sales of hookahs [17].

To our knowledge, this review is the first to take a comprehensive approach to identifying peer-reviewed journal articles published globally that examine the prevalence and main determinants of hookah smoking among youth. However, most of the displayed findings were generated from cross-sectional investigations [2-15,28-129] which renders it challenging to assess causality and make robust conclusions. Findings from various longitudinal studies, nevertheless, also confirmed the high prevalence of hookah smoking among youth [107,130,131,135,136] and its main predictors such as co-addiction [135,145,177], SES, low refusal self-efficacy [135,143], positive attitude, knowledge on hookah’s harm [130], and the male gender [135,178].

Another limitation is the use of self-reported measures to assess and determine the prevalence and predictors of hookah smoking among youth. Social desirability bias could have thus impacted the findings of these investigations. Various studies did not use standardized or validated tools to estimate the prevalence of hookah smoking [4,58,60,113,119,130].

Nevertheless, their findings were consistent with other investigations that utilized pre-tested and validated instruments [13,49,81,89]. Moreover, several studies used a convenience sample from a single academic institution to assess hookah smoking prevalence which might have impacted the generalizability of the findings [37,89,93,96,157,162].

Our scoping review has limitations as well. First, we did not communicate with relevant experts to incorporate data from unpublished manuscripts and thus our review might be subjected to publication bias [179]. In addition, since we lacked access to databases such as Web of Science, we might have missed several articles published on this topic. Yet, by following inclusive search strategies, we made extensive efforts to identify and include all relevant published studies.

Future studies should attempt to assess hookah use longitudinally and select participants randomly from the community rather than through a convenience sample from a particular academic institution. This will lead to more robust conclusions regarding the cause-and-effect relationships between the different predictors and hookah use and will improve external validity [180]. Future studies should utilize instruments that have been tested for reliability and validity to examine the prevalence and predictors of hookah smoking. Self-reported hookah use should be verified with biochemical measures. Additional research should also examine the effect of policy changes and advertisements on hookah use and access among youth [181].

## Conclusions

Hookah smoking is highly prevalent and has been rising in popularity among the youth worldwide. The scoping review showed that the main predictors of hookah use among youth include various intrapersonal and interpersonal factors such as the male gender and having friends and/or family members who smoke hookah, along with policy-level determinants including the lack of enforcement of prohibiting laws. Public health authorities, educators, and other stakeholders should implement educational interventions to enhance the knowledge level on hookah smoking’s harm and addiction and should target not only the individuals but also the family and the social environment. Strict regulations should be set to restrict access of youth to hookahs and for the production, distribution, marketing, and sales of hookahs.

## Appendices

**Table 2:** References for the determinants of hookah smoking among youth

Determinants	References
Age	[3,5,6,13,35,49,62,66,68-70,76,77,80-83,88,97,99,126,138,139,182]
Gender	[5,6,11,14,29,39,40,44,49-51,55,59,61,64,69,71-74,76-78,80,83,86,88-90,94,98,99,118,120,123,125,127,128,131,138,139,142,167,168,178,183]
SES	[6,28,29,33,39,69,71,76,85,98,110,124,125,127,141-143,151,168]
Geographic region	[2-9,13,14,36-38,40-67,71-78,81-85,98,100-123,130-135,138]
Other addictive habits/substance abuse	
Cigarette smoking	[3,8,34,39,40,44,54,57,59,61,67,71,77,81,87-89,94,99,100,109-111,119,122,123,127,132,135,136,139,142,143,146,151,152,155,178,184]
Alcohol	[6,12,32,35,40,59,80,109,123,132,139,142,144,145,178]
Marijuana	[87,89,109,123,132,142,146,178]
Illicit drug use	[123,142]
Knowledge	
Less harmful than cigarettes	[4,10,28,33,36-38,48,65,69,71,79,81,83,84,88,89,92,109,119,122,123,132,149-151,153,156,160,161,165,166,169,182,185,186]
Less addictive than other forms of smoking	[4,10,60,69,74,79,83,92,109,152-156,164,169]
Lack of knowledge on hookah harm	[10,11,15,28,32,48,60,62,66,68,70,72,73,75,79,80,84,87,88,93,96,97,106,113,117,120,130,139,149,151,153,155-157,160,164-166,187,188]
Relaxation, fatigue relief, sensation, and trend	
Relaxation and fatigue relief	[4,10,35,38,45,74,93,105,113,153,178]
Stress and anxiety management	[9,38,46,74,156]
Pleasure and sensation seeking	[2,35,46,124,136,150,153,156,178]
Perception as trendy	[38,74,91,111,155,157]
Flavor	[54,71,88,91,92,97,149,158-160]
Social environment	[2,8-11,13,15,28,32,36,37,39,40,43,45,46,48,49,55,57-60,62,66,68,71,73,77,79,80,82,83,86,87,92,94-97,100,105,109,110,113,114,120-122,124,127,135,143,144,149,150,152-156,158-161,163-165,167]
Social acceptability	[2,58,60,87,95,113,121,150,153-156,159,165]
No intention of quitting hookah smoking	[42,45,57,87,98,116,157,161]
Attitude	[6,42,48,72,76,80,97,102,108,116,121,130,139]
Low hookah refusal self-efficacy	[47,51,135,143,163]
Curiosity	[9,74,80,84,92,98,150,164]
Accessibility and smoking location	
Cafes	[10,13,37,57-59,65,71,85,123,128,152,159,161]
Hookah lounges	[36,73,112,122,123]
Friend’s house	[10,14,87,100]
Home	[9,10,43,48,65,100,159]
Accessibility	[13,58,88,123,150]
Policies/Ads	[86,98,143,160]
